# Development of a Healthy Dietary Habits Index for New Zealand Adults

**DOI:** 10.3390/nu9050454

**Published:** 2017-05-03

**Authors:** Jyh Eiin Wong, Jillian J. Haszard, Anna S. Howe, Winsome R. Parnell, Paula M. L. Skidmore

**Affiliations:** 1 School of Healthcare Sciences, Faculty of Health Sciences, Universiti Kebangsaan Malaysia, Kuala Lumpur 50300, Malaysia; wjeiin@ukm.edu.my; 2 Department of Human Nutrition, University of Otago, Dunedin 9054, New Zealand; jillian.haszard@otago.ac.nz (J.J.H.); winsome.parnell@otago.ac.nz (W.R.P.); 3 Department of General Practice and Primary Health Care, University of Auckland, Auckland 1142, New Zealand; a.howe@auckland.ac.nz

**Keywords:** diet quality, food habits, nutrient intake, New Zealand adults

## Abstract

Healthful dietary habits are individually associated with better nutrient intake and positive health outcomes; however, this information is rarely examined together to validate an indicator of diet quality. This study developed a 15-item Healthy Dietary Habits Index (HDHI) based on self-reported dietary habits information collected in the 2008/09 New Zealand Adult Nutrition Survey. The validity of HDHI as a diet quality index was examined in relation to sociodemographic factors, 24-diet recall derived nutrient intakes, and nutritional biomarkers in a representative sample of adults aged 19 years and above. Linear regression models were employed to determine associations between HDHI quintiles and energy-adjusted nutrient data and nutritional biomarkers. Significantly higher HDHI scores were found among women, older age groups, Non-Māori or Pacific ethnic groups, and less socioeconomically-deprived groups (all *p* < 0.001). Increasing quintiles of HDHI were associated with higher intakes of dietary fibre and seven micronutrients including calcium, iron, and vitamin C, and lower intakes of energy, macronutrients, sodium, zinc, vitamins B6 and B12. Associations in the expected directions were also found for urinary sodium, whole blood folate, serum and red blood cell folate, and plasma selenium (all *p* < 0.001). The present findings suggest that the HDHI is a valid measure of diet quality as it is capable of discerning quality of diets of subgroups and ranking nutrient intakes among NZ adults.

## 1. Introduction

Dietary quality, as a global marker of food choices, has received much attention in terms of how it relates to health, particularly with regard to chronic diseases, such as cardiovascular disease (CVD) and cancer [1]. The most commonly used dietary indices (the Healthy Eating Index (HEI), Alternative HEI, Mediterranean Diet and Dietary Approaches to Stop Hypertension (DASH) scores) have all been shown to be associated with significant reduced risk of all-cause, CVD and cancer mortality [1,2,3]. There is also a small, but growing body of literature investigating the relationship between diet quality indices (DQI) and successful ageing [4].

One potential advantage of using a DQI, would be that it can be derived from a short Food Frequency Questionnaire (FFQ) or other questionnaires. While this is the case in some studies [5,6,7], a large proportion of research involving dietary indices is based on nutrients and/or food components [1,8]. This requires the collection of comprehensive food intake data such as 24 h diet recalls or diet records. The collection of such in-depth dietary data is not feasible, financially or in terms of participant burden, for every study. Therefore, there is a need to develop a simpler tool to summarize diet quality, that involves a brief questionnaire, and that is specific to the population of interest.

Brief questionnaires such as dietary habits questionnaires are frequently used in large population studies [9,10] as these questions are easier to administer and readily interpretable. Instead of assessing food intake quantitatively, dietary habits questions broadly assess habitual intake of certain food groups, food preparation, cooking practices, eating patterns, and liking of certain foods or food groups which may indicate compliance with prevailing dietary guidelines. Healthful dietary habits are individually associated with better nutrient intake and positive health outcomes [11], however this information is rarely examined together as an indicator of diet quality.

We have previously developed a DQI for New Zealand (NZ) adolescents based on aggregated information from a dietary habits questionnaire and assessed its validity in a nationally representative sample of 15–18-year-old taking part in the NZ Adults Nutrition Survey (2008/09 NZANS). The index, namely Healthy Dietary Habits Score for Adolescents (HDHS-A) was found to have good validity [12], however we cannot assume that the HDHS-A would be valid for use in New Zealand adults, whose food choices differ from their younger counterparts [13]. Therefore, the aims of this study are to (1) develop a DQI specifically for use in adults; and (2) examine the validity of this index and associations with sociodemographic factors, nutrient intakes, and nutritional biomarkers in those aged 19 years and above who participated in the 2008/09 NZANS.

## 2. Materials and Methods

### 2.1. Study Design and Sample

This is a secondary analysis of adult participants aged 19 years and above from the 2008/09 NZANS, a cross-sectional population based survey in a representative sample of New Zealanders. Based on a multistage, stratified, probability-proportional-to-size sampling frame, 4742 home interviews were conducted in 2008–2009 among 4721 adults aged 15 years and above. Data from 3993 participants aged between 19 and 98 years were included in the present study. The study protocol of this survey was granted ethical approval (MEC/08/04/049) by the New Zealand Health and Disability Multi-Region Ethics Committee and described in detail elsewhere [14].

### 2.2. Dietary Intake

Dietary intake data were collected via multiple-pass 24-h diet recalls using structured interviews. Using the LINZ24© module of Abbey Research software package (LINZ^®^ Health and Activity Research Unit, University of Otago, Dunedin, New Zealand), participants were prompted to report all foods and drinks consumed in the preceding day (midnight to midnight) in four stages. First, participants reported a ‘quick list’ of all foods, beverages, and dietary supplements consumed, followed by detailed descriptions of their cooking methods, recipe (where applicable), brand and product name (where known), time and place of consumption. For food products where packaging was available, a bar code scanner was used to retrieve the brand and product name alongside their nutrient composition. Next, the amount consumed was estimated using portion estimation aids such as tablespoons, cups, shape dimensions, food photographs and food packaging information. Lastly, all items were reviewed in chronological order to clarify ambiguities and any addition and changes were made in this stage as necessary. A second 24-diet recall was conducted in a representative sub-sample (25%), however only the first 24-h diet recall data are used in present analyses. Nutrient intakes were calculated without including dietary supplements. This was done by matching the food and beverage information collected from the single 24-h diet recall to the New Zealand Food Composition database (FOODfiles), or the relevant overseas database, where applicable.

To complement the dietary information from 24-h dietary recall, each participant was also asked to complete a 25-item Dietary Habits Questionnaire (DHQ) [15]. This questionnaire comprises questions sourced from previously validated questionnaires and focuses on key dietary habits associated with diet quality and nutritional status. It has been tested for face validity using cognitive interviewing before use in the 2008/09 NZANS [14]. In addition to frequency questions focusing on previous four-week intakes of breakfast and selected food and food groups, there were questions on daily intake of fruit and vegetable servings and qualitative information on food preparation and cooking practices, and the use of low-fat and low-sodium foods (see Supplementary Table S1). All responses were recorded into a laptop computer using a computer-assisted personal interview software (Abbey Research Software, LINZ^®^ Health and Activity Research Unit, University of Otago, Dunedin, New Zealand). Participants aged 19 years and above who completed both single 24-h diet recalls and at least 75% of the Dietary Habits Questionnaire were included in the data analysis.

### 2.3. Demographic and Lifestyle Factors

Participants were asked to self-identify one or more ethnic groups to which they belonged. Using a hierarchical classification approach, three mutually exclusive ethnic groups were derived, namely Māori, Pacific, and Others. The latter comprises European alongside Asian, Middle Eastern, Latin American, and African ethnic groups.

A small-area measure of deprivation, NZ Deprivation Index (NZDep2006) was used to estimate relative socioeconomic status of participants. Based on eight dimensions of deprivation for each neighbourhood in New Zealand, the NZDep2006 decile categorization was collapsed to quintiles. Quintile 1 represents that a participant is living in the least deprived 20 percent of small areas, while Quintile 5 represents living in the most deprived 20 percent of small areas [16].

To assess habitual alcohol consumption, participants were asked whether they had ever consumed alcohol and about their alcohol consumption in the last 12 months. For those who indicated ‘Yes’ to past 12-month alcohol consumption, they were further prompted to report their consumption frequency and quantity (in number of drinks). This information was used to classify participants into four groups: non-drinkers, former drinkers, light to moderate drinkers and heavy drinkers (approximately >4 drinks/day for men and >2 drinks/day for women). Smoking status (non-smoker, former smoker, and current smoker) was self-reported by participants. Former smokers are defined as those who are not currently smoking but have smoked a total of more than 100 cigarettes in their entire life.

### 2.4. Anthropometric Measurements

Using standardised protocols, height was measured to the closest 0.1 cm using a portable stadiometer (Seca 214, Hamburg, Germany). Body weight was measured, with participants in light clothing, to the closest 0.1 kg using calibrated electronic weighing scales (Tanita HD-351, Tanita Corporation, Arlington Heights, IL, USA). Waist circumference was measured to the closest 0.1 cm at the end of a normal expiration and mid-point between the lower costal border and the iliac crest using a steel tape (W606PM, Lufkin, TX, USA). All measurements were taken in duplicate, and a third measure was taken if the first two measures differed by more than 1 percent. Body mass index (BMI) was calculated by dividing body weight in kilograms by the square of height in metres. (kg·m^−2^). Weight status was defined based on WHO BMI cut-offs: underweight (BMI < 18.5 kg·m^−2^), normal (BMI = 18.5–24.9 kg·m^−2^), overweight (BMI = 25.0–29.9 kg·m^−2^) and obese (BMI ≥ 30 kg·m^−2^).

### 2.5. Biochemical Assessments

A subsample consented to provide blood and spot urine samples within two weeks after the home interviews. Non-fasting blood was collected from a forearm vein into one 10 mL vacutainer without additive, and two 4 mL vacutainers containing ethylene diamine tetra-acetic acid (EDTA). All blood samples were stored at 4 degrees Celsius until analysed. The blood biomarkers of nutritional status examined in this study were whole blood folate, serum folate, red blood cell (RBC) folate, serum vitamin B12, and plasma selenium.

Whole-blood and serum folate was analysed using a microbiologic assay on 96-well microtiter plates with chloramphenicol-resistant *Lactobacillus casei* as the test microorganism. RBC folate was calculated by subtracting serum folate from whole-blood folate with correction for haematocrit [17]. Serum vitamin B12 was measured using a chemiluminescent kit on a Roche Hitachi Elecsys 2010 analyser (Roche Diagnostics, Basel, Switzerland). Plasma selenium concentrations were determined by graphite furnace Atomic Absorption Spectroscopy with Zeeman background correction (AA-800; Perkin-Elmer Corp, Norwalk, CT, USA) by a modification of the Jacobson and Lockitch’s method [18].

All urine samples were processed at the Canterbury Health Laboratories for urinary sodium, potassium and iodine. Urinary sodium and potassium were determined using an Abbott Architect C8000 analyser with an ion selective electrode (Abbott Diagnostics, Auckland, New Zealand), while urinary iodine was analysed using Sandell-Kolthoff colorimetric method [19]. Not every participant provided a blood or urine sample. From those participants providing samples, the amount of blood and urine obtained was not sufficient for conduction of all biochemical tests for everyone but, data were obtained for at least one nutrient from 2903 participants.

### 2.6. Scoring of the Healthy Dietary Habits Index (HDHI)

The items of HDHI were developed using information from the DHQ. Each item was scored from 0 to 4, whereby a higher score was assigned to a dietary habit response that aligned more closely to the New Zealand Food and Nutrition Guidelines for Healthy Adults (2003). An expert panel comprising nutritionists, a dietitian and statistician reviewed the index items and ensured that a maximum score of four was given to the healthiest response in terms of dietary habits for each item. Specifically, higher scores were assigned to food preparation, choices, or habits that result in lower total or saturated fat intake for items 1–7. For example, a full score of 4 was given for item 4 if a participant consumed ‘trim milk’ which has the lowest total fat content compared to other choices of milk. As milk is considered part of a healthy diet a zero score was given if no milk was regularly consumed. Items 8–10 concerned dietary fibre intake, therefore higher scores were given to consumption of wholegrain bread and compliance with the recommended servings for fruits and vegetables. Item 11 captures intake of added sugar from sugar-sweetened beverages, with higher scores indicating healthier beverage consumption habits with respect to total sugar intake. In addition, healthy dietary habits were defined by frequency of breakfast consumption and fast food consumption in items 12 and 13, respectively. Lastly, items 14–15 assessed salt habits, and gave points based on discretionary salt use (i.e., salt added at the table) and preference for low-salt products. Table 1 presents the items and scoring system of the HDHI.

### 2.7. Statistical Analysis

All analyses were carried out using the statistical software package Stata version 14.1 (StataCorp, College Station, TX, USA). To account for complex survey sampling design, survey prefix and sample weights were applied to produce estimates (means, medians, proportions) that represented the total resident population aged 19 years and over in New Zealand.

Structural Equation Modelling (SEM) was applied to the DHQ scores (from Supplementary Table S1) to select the *a-priori* index items. Items that loaded less than 0.25 were removed from the final index. The statistical fit of the HDHI was determined using two goodness of fit statistics: the standardized root mean square residual (SRMR) which should be less than 0.08 [20] and the coefficient of determination (CD) where 1 indicates perfect fit. For each participant, a total index score was then calculated by summing up scores from all index items, provided the participant had answered at least 75% (18 of 25) of the items from the DHQ. Missing items were imputed as the mean of the remaining items. Internal reliability of the HDHI was assessed using the Cronbach’s alpha and correlations.

To establish construct validity, we hypothesized that a higher HDHI score is (i) found in participants with higher socioeconomic status, non-smokers, and non-alcohol drinkers; (ii) associated with a more desirable nutrient profile; and (iii) associated with a more favourable nutritional biomarker profile. Only participants with complete 24-h diet recall data were included in the analyses. For the first hypothesis, linear regression was used to evaluate associations with demographic characteristics: sex, age group, ethnicity, deprivation, weight status, smoking status, and alcohol drinking status. Post-hoc pairwise comparisons used a Bonferroni multiple comparison adjustment, while age group and NZDep2006 were assessed by a trend analysis.

A total of 25 nutrient variables were derived from the 24 h recall data. They were energy, protein, fat (total, saturated, monounsaturated, polyunsaturated fats), total carbohydrate, sodium, dietary fibre, total sugar, sucrose, fructose, calcium, iodine, iron, potassium, selenium, zinc, beta-carotene, folate, vitamins A, B6, B12, C, and E. These nutrients were adjusted for energy intake using the Willett residual method [21]. HDHI scores were categorised by quintile into five groups (Q1–Q5) and a trend analysis across groups for each nutrient was undertaken using linear regression.

Finally, to test the third hypothesis, similar linear regression models were undertaken using HDHI quintiles as the independent variable and nutritional biomarkers as the dependent variable. All blood and urinary biomarker variables were log-transformed due to positive skew and geometric means with 95% confidence intervals are presented. Because supplement use could confound the relationship between the index score and biomarkers [22], an interaction term was included in the regression model between HDHI quintiles and self-reported supplement use in the last 12 months (yes/no). When an interaction was significant (*p* < 0.05), a sensitivity analysis was carried out only in participants who were not supplement users.

## 3. Results

### 3.1. Sample Characteristics

A total of 3993 adults (1729 males, 2264 females) aged 19 to 98 years who completed at least 75% of the DHQ were included in the initial analysis to construct the diet index. Missing data (1.6% of total data points) were imputed using mean of the non-missing data for a variable in the calculation of the HDHI score.

### 3.2. Internal Reliability of the HDHI

Out of the initial 20 items, five dietary habits items with factor loadings of less than 0.25 on the first factor were dropped (processed meat, types of cooking fat, fruit juice, confectionery, and iodised salt). The loading cut-off of 0.25 was chosen because anything higher would have excluded vegetable intake (loading = 0.28), which was considered an important component of a healthy diet [23], and all items that loaded less than this (and were therefore excluded) loaded at very low levels (all less than 0.20). The final diet index consists of 15 items with a possible score ranging from 0 to 60 (Table 1).

The 15 items represented intake of recommended food group servings (fruits and vegetables), intake frequency of non-core food groups (fries, soft drinks, fast foods), food preparation habits (fat trimming for meat and chicken), food preferences (types of bread, milk, fat spread, fish), use of healthier food products (low-fat and low-salt variety), breakfast and salt habits. The SRMR was 0.063 while the CD was 0.78, indicating that the HDHI had acceptable fit. The correlations of each item with the total score ranged from 0.35 to 0.65, highlighting that item 6 (i.e., use of low-fat products) had the greatest influence on the index. Overall, the HDHI had good internal reliability with Cronbach’s alpha coefficient of 0.76 (Table 2).

### 3.3. Association with Sociodemographic and Lifestyle Factors

The average HDHI score was 40.4 (SE 0.2), with women scoring significantly higher than men (*p* < 0.001). As shown in Table 3, a higher HDHI score was associated with an older age group (*p* < 0.001), ethnic groups other than Māori and Pacific (*p* < 0.001), lower NZDep2006 Quintile (*p* < 0.001), normal or overweight groups (*p* < 0.001), former and non-smokers (*p* < 0.001), and non-alcohol drinkers (*p* < 0.001).

### 3.4. Association with Nutrient Intakes and Nutritional Biomarkers

Table 4 and Table 5 present the associations of HDHI with nutrient and nutritional biomarker levels, respectively. Univariate linear regression analyses showed that lower intakes of energy, protein, total fats (including saturated and monounsaturated fats), total carbohydrate, sodium, sucrose, zinc, vitamins B6 and B12 were associated with increasing quintiles of HDHI. Conversely, higher intakes of dietary fibre, fructose, calcium, iron, potassium, beta-carotene, folate, vitamins C, and E were associated with increasing HDHI scores (Table 4). Associations in the expected directions were also found for all nutritional biomarkers, except for urinary iodine (*p* = 0.076) and serum B12 (*p* = 0.264), which showed no significant trend. Supplement use was found to be a significant moderator in the association with urinary sodium (*p* = 0.025), whole blood folate (*p* < 0.001), red blood cell folate (*p* = 0.002), and serum B12 (*p* = 0.042), therefore sensitivity analyses were undertaken using only participants who were not taking dietary supplements and compared to the whole sample trends. Reduction of the sample size resulted in mildly attenuated, but still significant, trends for all biomarkers except for serum B12, whereby non-supplement users had a trend of lower serum B12 levels across the quintiles of HDHI scores (*p* = 0.001), which was not seen in the full biomarker sample (*p* = 0.264) (Table 5).

## 4. Discussion

This study addressed the critical need for a simple yet valid diet index to assess the diet quality of NZ adults. We developed a 15-item diet index namely HDHI based on dietary habits information and validated the index against information from 24-h diet recall and nutritional biomarkers in a representative sample of NZ adults.

### 4.1. HDHI vs. Sociodemographic and Lifestyle Factors

The HDHI reported excellent internal validity and was able to distinguish differences in diet quality between groups of participants with different sociodemographic and lifestyle factors hypothesized to have diverging diet quality. The mean HDHI scores were highest among women, older age groups, Non-Māori or Pacific ethnic groups, and less socioecomically-deprived groups. In line with literature [24,25], diet quality was found to follow a socioeconomic gradient in this study, as the mean HDHI increases with higher socioeconomic status (*p*-trend < 0.001). Akin to this, it was observed that the mean HDHI scores of Māori and Pacific ethnic groups were approximately 15% lower than their non-Māori, non-Pacific counterparts. This finding is likely linked to socioeconomic disparities among ethnic groups in New Zealand [26], as a disproportionately higher number of Māori (72%) and Pacific participants (81%) were also reported to live in the two most deprived areas (NZDep2006 Quintiles 4 and 5) compared with 41% of Others.

While older adults tend to have better dietary status than younger adults [27], our findings of the oldest adult group (aged 71 years and older) having the highest HDHI scores contradict with our understanding that diet quality declines gradually in the ninth decade of life [28]. This may reflect a cohort effect, whereby older participants for imminent health reasons, are more likely to choose healthier varieties of foods or products (e.g., low-fat milk, spread, low-salt foods) that are given higher scores in the HDHI. It may also reflect the ability of the HDHI in capturing diet based on food selection, variety and other qualities, rather than quantity and energy.

Concurring with previous literature [29,30], this study found that obese adults had less healthy diets than normal- and overweight adults, as indicated by lower HDHI scores. Poor-quality diets also tend to be clustered with unhealthy lifestyles such as smoking and alcohol binging. Former and non-smokers reported better diet quality as shown by higher HDHI scores compared to current smokers, and this finding corroborates findings from previous studies [31,32,33]. Alcohol consumption appears to be related to HDHI scores, as heavy drinkers scored lowest in HDHI, followed by former drinkers and non-drinkers. For light/moderate drinkers, their HDHI scores were reported to be similar to former and non-drinkers (*p* > 0.05), which supports the notion that moderate alcohol consumption (up to 4 drinks/day for men and 2 drinks/day for women) can be part of a healthy diet.

### 4.2. HDHI vs. Nutrient Intake Levels

When compared with data from 24-h diet recalls, higher HDHI scores were significantly associated with energy-adjusted intakes of key nutrients in the expected directions. Individuals in higher quintiles of the HDHI had higher intakes of dietary fibre, fructose, calcium, iron, potassium, beta-carotene, folate, and vitamins C and E. Additionally, higher HDHI scores were associated with lower intakes of all macronutrients (carbohydrate, protein, total fat, saturated and monounsaturated fats), as well as sodium and sucrose.

Notably, there were large differences between participants in the lowest (Q1) and highest (Q5) quintiles of the index with regards to intakes of total fat and sucrose. Total fat and sucrose intakes were lower by 26% (mean difference 22 g) and 30% (mean difference 18 g), respectively from the highest quintile to the lowest quintile of the HDHI scores. The efficacy of HDHI in capturing total fat intake was not unexpected, as seven out of the 15 items of the HDHI focuses on food preparation, preference and habits related to total fat intakes (see Table 1). For sucrose intake, the main sources of sucrose in NZ adults’ diets are sugar and sweets, followed by non-alcoholic beverages, and fruits [13]. Interestingly, despite only one clear indicator with added sucrose being used in the HDHI, the item ‘soft drink or energy drink consumption’ appears to have successfully captured the variation in sucrose intake for this sample. In addition, protein, zinc, and vitamins B6 and 12 were lower for participants with the highest quintiles of HDHI scores compared to those with the lowest scores. This inverse association is to be expected as food items that are high in these nutrients (e.g., meat, potatoes and kumara, white bread) were scored lower in the HDHI. Taken together, these results confirm that the HDHI reflects the intake of a variety of nutrients indicative of diet quality.

### 4.3. HDHI vs. Nutritional Biomarkers

This study also found that higher HDHI scores were associated with better nutritional biomarker levels. Consistent with findings from dietary recall data, lower levels of urinary sodium, and higher levels of whole blood folate, serum folate and red blood cell folates, and plasma selenium were found to be associated with increasing HDHI scores. Associations were attenuated but still remained for those individuals who reported no supplement use within the previous 12 months for urinary sodium, whole blood and red blood cell folate.

Contradictory findings for dietary and urinary potassium were seen in the current study as increases in HDHI scores were associated with increasing dietary potassium and decreasing urinary potassium. In this study, only a spot urine sample was collected between 2 to 3 weeks after the home interviews. Therefore day-to-day and diurnal variations in potassium excretion [34] were not accounted for, and this may have confounded the results of the current study.

### 4.4. Strengths and Weaknesses

A key strength of this study lies in the simplicity of the HDHI which is easy to administer and does not require conversion of foods to nutrient content in its scoring. As a summary measure, HDHI examines various aspects of dietary habits that are important indicators of diet quality, including food preparation habits, food preferences, use of healthier food products, breakfast and salt habits, and intake of recommended and non-core food groups. Input from panel members whose expertise is in dietary assessment in the NZ nutrition surveys were sought to ensure face and content validity of the HDHI. Besides internal validity, construct validity was examined using independent reference measures of 24-h diet recalls and nutritional biomarkers. This consideration of a broad definition of validity added credence to the robustness of the validity results for the diet index. Another important strength of this study is in the use of a nationally representative sample from the 2008/09 NZANS to develop and validate this diet index. The validity of the HDHI should therefore be generalizable to the wider population of NZ adults aged 19 years and above.

Finally, a number of important limitations need to be noted regarding this study. First, the use of single 24-h diet recall data provides a snapshot of the population’s nutrient intake, however it does not take into account day-to-day variation in nutrient intakes which may have attenuated the HDHI-nutrient relationships in the regression models. Second, the reliance of self-reported dietary habits is also subjected to social desirability bias and may not truly reflect what is actually eaten. Third, information on physical activity was not collected in the 2008/09 NZANS which precluded the examination of physical activity in association with HDHI. Lastly, while the HDHI is capable of discriminating against those in the lowest and in the highest quintiles, there is no quantitative cut-offs or benchmark to define a ‘healthy’ diet. Therefore, this index may only be useful to rank diet quality in a population. Deserving further examination, is the validity of the HDHI in predicting positive health outcomes. A way forward is to establish the predictive performance of this diet index by examining this index prospectively in relation to health and related outcomes or mortality risks.

## 5. Conclusions

The present findings suggest that the HDHI has the ability to discern quality of diets of subgroups in NZ population and indirectly assess the intakes of nutrient intakes. Based on dietary habits information, HDHI is a valid summary measure of total diet and serves as a practical way to assess diet quality among NZ adults. Further research is recommended to validate the HDHI in a longitudinal study of nutrition and health outcomes.

## Figures and Tables

**Table 1:** Items and scoring of the Healthy Dietary Habits Index (HDHI).

Items	Description ^1^	Scoring Criteria ^2^				
		0 (Less Healthy)	1	2	3	4 (More Healthy)
1 (Red Meat)	Trimming meat fat before consumption	Never	Rarely	Sometimes	Regularly	Always
2 (Chicken)	Trimming chicken fat before consumption	Never	Rarely	Sometimes	Regularly	Always
3 (Fish/shellfish)	Proportion of fried fish/shellfish relative to total fish/shellfish intake	Never consume fish/shellfish	100%–76%	75%–51%	26%–50%	0%–25%
4 (Milk)	Types of milk consumed	None	Whole or standard milk	Other (e.g., rice, goats milk)	Reduced fat /Soy milk	Skim or trim milk
5 (Spread)	Types of fat spread used	Butter	Butter & margarine blend	Margarine ^3^ (full fat)	Plant sterol margarine ^4^	None/Margarine (light or reduced fat)
6 (Low-fat foods)	Use of low-fat products	Never	Rarely	Sometimes	Regularly	Always
7 (Fries)	Intake of potatoes and kumara ^5^ fries per week	7 or more times/week	5–6 times/week	3–4 times/week	1–2 times/week	Never, <1/week
8 (Bread)	Types of bread consumed	Don’t eat bread	White/Other		Light grain ^6^/High fibre white bread	Heavy grain bread ^7^
9 (Fruit)	Fruit intake per day	Never	Less than one serving	1 serving		2–4 servings
10 (Vegetables)	Vegetables intake per day	Never	Less than one serving	1 serving	2 servings	3 or more servings
11 (Soft drinks)	Soft drink or energy drink consumption per week	7 or more times/week	5–6 times/week	3–4 times/week	1–2 times/week	Never, <1/week
12 (Breakfast)	Breakfast consumption per week	0	1–2	3–4	5–6	7
13 (Fast foods)	Purchasing fast food or takeaways	7 or more times/week	5–6 times/week	3–4 times/week	1–2 times/week	Never, <1/week
14 (Added salt)	Adding salt to foods before eating	Always	Regularly	Sometimes	Rarely	Never
15 (Low-salt foods)	Use of low-salt products	Never	Rarely	Sometimes	Regularly	Always

^1^ Refer to Supplementary Table S1 for original questions from the Dietary Habits Questionnaire; ^2^ Scoring criteria based on responses from the Dietary Habits Questionnaire; ^3^ Fat spread made from vegetable oils such as canola, sunflower and olive oils; ^4^ Margarine spread containing phytosterols, including both full and low-fat varieties such as Proactive and Logicol; ^5^ Sweet potatoes; ^6^ Including commercial brands such as Molenberg, Freya’s, Ploughmans, and MacKenzie High Country; ^7^ Including commercial brands such as Vogels and Burgen.

**Table 2:** Internal reliability of index items (*n* = 3993).

Item	*n* ^1^	Item-Test Correlation	Cronbach’s Alpha Coefficient
1. Red meat	3760	0.60	0.74
2. Chicken	3649	0.60	0.74
3. Fish/shellfish	3987	0.38	0.77
4. Milk	3983	0.55	0.74
5. Spread	3979	0.41	0.76
6. Low-fat foods	3927	0.65	0.73
7. Fries	3991	0.45	0.75
8. Bread	3913	0.51	0.75
9. Fruit	3989	0.48	0.75
10. Vegetables	3988	0.35	0.76
11. Soft drinks	3987	0.47	0.75
12. Breakfast	3992	0.53	0.75
13. Fast food	3992	0.44	0.75
14. Added salt	3991	0.42	0.76
15. Low-salt foods	3818	0.50	0.75
Total HDHI score			0.76

^1^ Participants who had completed at least 75% of the DHQ questions without imputed data.

**Table 3:** Association between HDHI and sociodemographic factors.

Variables	*n* (%)	Mean HDHI Score (SE) ^1^	*p*-Value ^2^
Total ^3^	3993	40.4 (0.2)	
Sex			<0.001
Male	1729 (43)	38.5 (0.3)	
Female	2264 (57)	42.2 (0.3)	
Age group			<0.001 ^3^
19–30 years	716 (18)	36.9 (0.5)	
31–50 years	1338 (34)	39.5 (0.3)	
51–70 years	887 (22)	42.9 (0.4)	
71 years and over	1052 (26)	44.3 (0.3)	
Ethnicity			<0.001
Maori	921 (23)	35.0 (0.5) ^a^	
Pacific	633 (16)	35.8 (0.4) ^a^	
Other	2439 (61)	41.3 (0.2) ^b^	
NZDep2006 Quintile			<0.001 ^3^
1	524 (13)	42.6 (0.5)	
2	667 (17)	41.6 (0.5)	
3	634 (16)	41.1 (0.5)	
4	929 (23)	39.1 (0.4)	
5	1239 (31)	37.4 (0.5)	
Weight status			<0.001
Underweight	39 (1)	39.2 (2.5) ^c,d^	
Normal weight	943 (25)	41.1 (0.4) ^c^	
Overweight	1408 (37)	41.0 (0.3) ^c^	
Obese	1425 (37)	39.0 (0.4) ^d^	
Smoking			<0.001
Never	1786 (45)	42.3 (0.3) ^e^	
Current	974 (24)	34.1 (0.4) ^f^	
Former	1233 (31)	41.8 (0.4) ^e^	
Alcohol drinking			<0.001
Non-drinker	240 (6)	42.3 (0.7) ^g^	
Former drinker	532 (13)	39.6 (0.6) ^h^	
Light/moderate drinker	2869 (72)	40.9 (0.2) ^g,h^	
Heavy drinker	352 (9)	36.5 (0.8) ^i^	

^1^ Weighted for survey design; ^2^ Unadjusted regression models with HDHI as the dependent variable; ^3^ Trend tests for age group and NZDep2006 Quintile; ^3^ Only includes those that had answered at least 75% of the items and therefore had a HDHI score calculated; ^a–i^ Mean HDHI scores with unlike superscript letters indicate significant differences between groups after Bonferroni adjustment (*p* < 0.05).

**Table 4:** Association between HDHI and dietary intake (*n* = 3993).

Nutrients	HDHI Q1 Mean (95% CI)	HDHI Q3 Mean (95% CI)	HDHI Q5 Mean (95% CI)	*p*-Trend
HDHI score	26 (25.8, 26.6)	40 (40.0, 40.3)	52 (51.6, 52.0)	-
Energy (MJ)	11 (10.3, 11.4)	9 (8.7, 9.6)	8 (7.7, 8.4)	<0.001
Protein (g)	85 (81, 90)	84 (81, 87)	81 (80, 84)	0.027
Total fat (g)	86 (83, 90)	78 (75, 80)	64 (62, 67)	<0.001
Total carbohydrate (g)	241 (233, 250)	236 (229, 243)	227 (220, 234)	0.004
Sodium (g)	2.7 (2.5, 2.8)	2.3 (2.2, 2.5)	2.2 (2.1, 2.3)	<0.001
Dietary fibre (g)	19 (18, 19)	23 (22, 24)	26 (25, 27)	<0.001
Saturated fat (g)	36 (34, 37)	30 (29, 32)	23 (22, 24)	<0.001
Monounsaturated fat (g)	32 (30, 33)	29 (27, 30)	24 (23, 25)	<0.001
Polyunsaturated fat (g)	10.8 (10.2, 11.4)	10.9 (10.4, 11.4)	10.9 (10.4, 11.5)	0.442
Fructose (g)	18 (16, 19)	20 (18, 21)	22 (21, 23)	<0.001
Sucrose (g)	60 (55, 65)	48 (45, 51)	42 (39, 45)	<0.001
Total sugars (g)	114 (107, 120)	106 (101, 111)	105 (101, 109)	0.069
Calcium (mg)	758 (710, 805)	840 (792, 889)	927 (878, 976)	<0.001
Iodine (μg)	61 (56, 66)	64 (57, 71)	58 (55, 61)	0.240
Iron (mg)	10.1 (9.6, 10.6)	11.8 (11.3, 12.3)	11.9 (11.4, 12.3)	<0.001
Potassium (g)	2.8 (2.7, 2.9)	3.1 (3.0, 3.2)	3.3 (3.2, 3.4)	<0.001
Selenium (mg)	57 (52, 61)	59 (53, 65)	56 (52, 60)	0.642
Zinc (mg)	10.7 (10.2, 11.3)	10.7 (10.3, 11.2)	10.3 (9.9, 10.6)	0.018
Beta-carotene equivalents (mg)	2.0 (1.7, 2.2)	2.8 (2.5, 3.2)	3.4 (2.9, 4.0)	<0.001
Total vitamin A (μg)	720 (665, 773)	890 (785, 995)	856 (783, 969)	0.078
Vitamin B6 (mg)	2.7 (2.2, 3.2)	1.9 (1.7, 2.1)	1.9 (1.7, 2.0)	0.002
Dietary folate equivalents (μg)	269 (251, 278)	363 (340, 387)	396 (372, 420)	<0.001
Vitamin B12 (mg)	4.8 (4.2, 5.4)	4.4 (3.9, 5.0)	3.6 (3.3, 3.9)	<0.001
Vitamin C (mg)	76 (68, 85)	107 (97, 117)	125 (115, 134)	<0.001
Vitamin E (mg)	9.3 (8.8,9.8)	10.5 (10.1, 10.9)	10.7 (10.2, 11.1)	<0.001

**Table 5:** Association between HDHI and nutritional biomarker levels.

Biomarker	*n*	HDHI Q1 Mean (95% CI)	HDHI Q3 Mean (95% CI)	HDHI Q5 Mean (95% CI)	*p*-Trend
Urinary iodine (µmol/L)	2871	0.56 (0.51, 0.61)	0.57 (0.47, 0.67)	0.50 (0.44, 0.56)	0.076
Urinary sodium (mmol/L)	2893	103 (96, 111)	83 (77, 89)	57 (53, 61)	<0.001 ^1^
Urinary potassium (mmol/L)	2893	61 (56, 66)	59 (55, 63)	50 (47, 54)	<0.001
Whole blood folate (nmol/L)	2558	305 (286, 326)	367 (348, 387)	407 (387, 429)	<0.001 ^2^
Serum folate (nmol/L)	2852	17 (15, 18)	23 (22, 25)	30 (28, 33)	<0.001
RBC folate (nmol/L)	2462	672 (630, 718)	837 (794, 914)	943 (895, 994)	<0.001 ^3^
Serum B12 (pg/mL)	2640	332 (314, 351)	302 (289, 317)	322 (308, 337)	0.264 ^4^
Plasma selenium (µg/L)	2526	90 (87, 93)	97 (94, 99)	101 (99, 104)	<0.001

^1^ Trend was the same for urinary sodium but attenuated for those not on supplements in the last 12 months (*n* = 1641, *p* = 0.025); ^2^ Trend was the same for whole blood folate but attenuated for those not on supplements in the last 12 months (*n* = 1459, *p* < 0.001); ^3^ Trend was the same for RBC folate but attenuated for those not on supplements in the last 12 months (1397, *p* = 0.002); ^4^ Negative trend found for serum B12 for those not taking supplements in the last 12 months (*n* = 1488, *p* = 0.042): Q1: 333 (311, 355); Q3: 298 (280, 316); Q5: 283 (267, 299), *p*-for-trend = 0.001.

## Supplementary Materials

The following are available online at http://www.mdpi.com/2072-6643/9/5/454/s1, Table S1: Original questions in the Dietary Habits Questionnaire and scoring of the Healthy Dietary Habits Index (HDHI).
