# Supplementary material for: Development of a Healthy Dietary Habits Index for New Zealand Adults
Source: Nutrients. 2017 May 3;9(5):454. doi: 10.3390/nu9050454 (PMC5452184; doi:10.3390/nu9050454)
Supplement: Supplementary file 1 [file nutrients-09-00454-s001.pdf]

Supplementary

Table S1. Original questions in the Dietary Habits Questionnaire and scoring of the Healthy Dietary Habits Index (HDHI).

Items	Description	Original Question(s)[1]	Scoring Criteria[2]				
			0 (less healthy)	1	2	3	4 (more healthy)
1 (Red Meat)	Trimming meat fat before consumption	In the past four weeks, have you eaten red meat?	Yes	Yes	Yes	Yes	No
		If yes, how often do you remove excess fat from meat?	Never	Rarely	Sometimes	Regularly	Always
2 (Chicken)	Trimming chicken fat before consumption	In the past four weeks, have you eaten chicken?	Yes	Yes	Yes	Yes	No
		If yes, how often do you remove the skin from chicken?	Never	Rarely	Sometimes	Regularly	Always
3 (Fish/shellfish)	Proportion of fried fish/shellfish relative to total fish/shellfish intake	How often do you eat fresh or frozen fish or shellfish? Do not include battered / fried or canned fish or shellfish<br>How often do you eat battered or fried fish or shellfish?<br>How often do you eat canned fish or shellfish	Never consume fish/shellfish	100-76%	75-51%	26-50%	0-25%
4 (Milk)	Types of milk consumed	What type of milk do you use the most of?	None	Whole or standard milk	Other (e.g. rice, goats milk)	Reduced fat / Soy milk	Skim or trim milk
5 (Spread)	Types of fat spread used	What type of butter or margarine spread do you use the most of?	Butter	Butter & margarine blend	Margarine[3] (full fat)	Plant sterol margarine[4]	None / Margarine (light or reduced fat)

Items	Description	Original Question(s)[1]	Scoring Criteria[2]				
			**0 (less healthy)**	**1**	**2**	**3**	**4 (more healthy)**
6 (Low-fat foods)	Use of low-fat products	How often do you choose low or reduced fat varieties of food instead of the standard variety?	Never	Rarely	Sometimes	Regularly	Always
7 (Fries)	Intake of potatoes and kumara fries per week	How often do you eat hot chips, French fries, wedges, or kumara[5] chips?	7 or more times/week	5-6 times/week	3-4 times/week	1-2 times/week	Never, <1/week
8 (Bread)	Types of bread consumed	On average, how many slices of bread/toast or bread rolls do you eat per day? What type of bread, rolls or toast do you eat most of?	Don't eat bread	White / Other		Light grain[6] / High fiber white bread	Heavy grain bread[7]
9 (Fruit)	Fruit intake per day	On average how many servings of fruit – fresh, frozen, canned or stewed - do you eat per day?	Never	Less than one serving	1 serving		2 servings, 3 servings, 4 servings
10 (Vegetables)	Vegetables intake per day	On average how many servings of vegetables – fresh, frozen or canned – do you eat per day?	Never	Less than one serving	1 serving	2 servings	3 servings, 4 or more servings
11 (Soft drinks)	Soft drink or energy drink consumption per week	How often do you drink soft drinks or energy drinks[8]?	7 or more times/week	5-6 times/week	3-4 times/week	1-2 times/week	Never, <1/week
12 (Breakfast)	Breakfast consumption per week	How many days in an average week do you have something to eat for breakfast?	0	1-2	3-4	5-6	7

Items	Description	Original Question(s)[1]	Scoring Criteria[2]				
			0 (less healthy)	1	2	3	4 (more healthy)
13 (Fast foods)	Purchasing fast food or takeaways	How often do you eat fast food or takeaways from places like McD, KFC, Burger King, Pizza shops or Fish 'n' Chip shops?	7 or more times/week	5-6 times/week	3-4 times/week	1-2 times/week	Never, <1/week
14 (Added salt)	Adding salt to foods before eating	How often do you add salt to your food after it has been cooked or prepared?	Always	Regularly	Sometimes	Rarely	Never
15 (Low-salt foods)	Use of low-salt products	How often do you choose low or reduced salt varieties of foods instead of standard variety?	Never	Rarely	Sometimes	Regularly	Always
(excluded)[9]	Fruit juice or drink consumption per week	How often do you drink fruit juices and drinks?	7 or more times/week	5-6 times/week	3-4 times/week	1-2 times/week	Never, <1/week
(excluded)[9]	Confectionery consumption per week	How often do you eat lollies, sweets, chocolate and confectionery?	7 or more times/week	5-6 times/week	3-4 times/week	1-2 times/week	Never, <1/week
(excluded)[9]	Processed meat intake per month	In the past four weeks, have you eaten processed meat[8]? If yes, how often do you eat processed meat products[10]?	7 or more times	5-6 times	3-4 times	1-2 times	<1 time, Never
(excluded)[9]	Types of cooking fat used	What type of fat or oil do you use most often when cooking?	Lard / Dripping	Butter	Butter blend	Margarine	None / oil
(excluded)[9]	Fruit juice or drink	How often do you drink fruit juices and drinks?	7 or more times/week	5-6 times/week	3-4 times/week	1-2 times/week	Never,

Items	Description	Original Question(s)[1]	Scoring Criteria[2]				
			**0 (less healthy)**	**1**	**2**	**3**	**4 (more healthy)**
	consumption per week						<1/week

[1] Original questions from the Dietary Habits Questionnaire.

[2] Scoring criteria based on responses from the Dietary Habits Questionnaire.

[3] Fat spread made from vegetable oils such as canola, sunflower and olive oils.

[4] Margarine spread containing phytosterols, including both full and low-fat varieties such as Proactive and Logicol.

[5] Sweet potatoes

[6] Including commercial brands such as Molenberg, Freya's, Ploughmans, and MacKenzie High Country.

[7] Including commercial brands such as Vogels and Burgen.

[8] Sugar-sweetened beverages excluding diet varieties.

[9] This item was not included in the final index.

[10] Including ham, bacon, sausages, luncheon, canned corned beef, pastrami and salami.
